# Genetic counselling and testing for inherited dementia: single-centre evaluation of the consensus Italian DIAfN protocol

**DOI:** 10.1186/s13195-020-00720-4

**Published:** 2020-11-17

**Authors:** Anna Mega, Samantha Galluzzi, Cristian Bonvicini, Silvia Fostinelli, Massimo Gennarelli, Cristina Geroldi, Orazio Zanetti, Luisa Benussi, Emilio Di Maria, Giovanni B. Frisoni

**Affiliations:** 1 Laboratory Alzheimer’s Neuroimaging & Epidemiology, IRCCS Istituto Centro San Giovanni di Dio Fatebenefratelli, Brescia, Italy; 2 Molecular Markers Laboratory, IRCCS Istituto Centro San Giovanni di Dio Fatebenefratelli, Brescia, Italy; 3 Genetics Unit, IRCCS Istituto Centro San Giovanni di Dio Fatebenefratelli, Brescia, Italy; 4 Department of Molecular and Translational Medicine, University of Brescia, Brescia, Italy; 5 Alzheimer’s Unit - Memory Clinic, IRCCS Istituto Centro San Giovanni di Dio Fatebenefratelli, Brescia, Italy; 6 Department of Health Sciences, University of Genoa, Genoa, Italy; 7 Unit of Medical Genetics, Galliera Hospital, Genoa, Italy; 8 University Hospitals and University of Geneva, Geneva, Switzerland

**Keywords:** Dementia, Alzheimer’s disease, Frontotemporal dementia, Genetic counselling, Genetic testing

## Abstract

**Background:**

A consensus protocol for genetic counselling and testing of familial dementia, the Italian Dominantly Inherited Alzheimer’s and Frontotemporal Network (IT-DIAfN) protocol, has been developed in Italy by a network of expert dementia centres. The aim of this study is to evaluate feasibility and acceptability of the genetic counselling and testing process, as undertaken according to the IT-DIAfN protocol in one of the IT-DIAfN dementia research centres.

**Methods:**

The protocol was tested by a multidisciplinary team at the IRCCS Istituto Centro San Giovanni di Dio Fatebenefratelli, Brescia, Italy, on affected individuals with suspected inherited forms of Alzheimer’s disease (AD) or frontotemporal dementia (FTD), and to healthy at-risk relatives. The genetic counselling and testing process consisted of (i) pre-test consultation and psychological assessment (ii) genetic testing, (iii) genetic test result disclosure and (iv) follow-up consultation and psychological assessment.

**Results:**

Twenty affected individuals from 17 families fulfilled the family history criteria of the IT-DIAfN protocol for suspected inherited dementia (17 for AD, 2 for FTD, 1 for inclusion body myopathy with Paget disease of bone and frontotemporal dementia) and were included in the protocol. Nineteen out of 20 affected individuals received the genetic test result (one left after the pre-test consultation being not ready to cope with an unfavourable outcome). A pathogenic mutation was found in 6 affected individuals (1 in *PSEN1*, 2 in *PSEN2*, 1 in *GRN*, 1 in *MAPT*, 1 in *VCP*). Eleven healthy at-risk relatives asked to undergo predictive testing and were included in the protocol. Three completed the protocol, including follow-up; one did not ask for the genetic test result after genetic testing; and eight withdrew before the genetic testing, mainly due to an increased awareness about the possible consequences of an unfavourable test result. To date, no catastrophic reactions were reported at the follow-up.

**Conclusions:**

Our case series shows that a structured genetic counselling and testing protocol for inherited dementia can be implemented in both affected individuals and at-risk relatives in a research setting. The procedure was shown to be safe in terms of occurrence of catastrophic events. A formal validation in larger cohorts is needed.

## Introduction

Alzheimer’s disease (AD) is the most common form of dementia and accounts for 60–70% of all dementia cases [1]. Frontotemporal dementia (FTD) represents an estimated 10–20% of all dementia cases and one of the most common presenile dementias [2]. In fewer than 5% of AD cases [3] and about 10% of FTD cases [4], these diseases are inherited as an autosomal dominant trait, by mutations in presenilin-1 (*PSEN1*), presenilin-2 (*PSEN2*) and the amyloid precursor protein (*APP*) genes, for AD [3], and microtubule-associated protein tau (*MAPT*), granulin (*GRN*) and chromosome 9 open reading frame 72 (*C9orf72*) genes, for FTD [5].

Although inherited AD and FTD represent a minority of all dementia cases, they constitute a critically important area of study, because the pathological features of genetic forms are similar to the more common sporadic ones, and research in the field contributes to advances in the basic scientific understanding of these diseases. Moreover, families with inherited dementia represent an ideal population for clinical trials with putative disease-modifying drugs, which have the potential to delay or even prevent dementia in asymptomatic individuals, in addition to slowing progression in those with symptoms.

The identification of deterministic genes associated to inherited AD and FTD enables members of families with a positive history for dementia to consider to undergo the relevant genetic test. When the family history is suggestive for inherited dementia, it is important to allow the patient and the family to understand the genetic risk and the possible options [6]. Being a carrier of a genetic variant causing familial dementia implies a risk for the relatives, especially for the offspring, with a remarkable burden also in spouses. When a disease-associated mutation is identified in a family, the consequences are not limited to the psychological outcomes caused by the unfavourable genetic test result. Finding an inherited form of dementia in a family impacts on all family members, including those who would have chosen not to know about the genetically determined risk [7]. Moreover, the disclosure of a genetically determined disease in a family is expected to raise ethical, legal and social issues [6]. In fact, the assessment of any genetic test, in order to be transferred in the clinical practice, should include safety and utility as well as the users’ perspective [8, 9].

A limited number of studies addressed genetic counselling and testing for inherited dementia (namely AD and FTD), as compared to the large body of literature focussed on Huntington’s disease (HD) and, more recently, amyotrophic lateral sclerosis (ALS). As recommended by the current guidelines for AD and the other inherited dementias [10], genetic counselling should always precede genetic testing to avoid negative outcomes, in both affected individuals and healthy at-risk relatives. However, a handful of papers addressed genetic counselling and testing for dementia to date. In the 5 years’ experience of a genetic counselling program in Spain, genetic testing was performed in 87 affected individuals from 72 families with suspected familial AD, FTD or prion disease, and in 23 at-risk relatives from the 22 families with an identified pathogenic mutation [11]. The program implemented a structured genetic counselling protocol only for at-risk relatives [11]. The other published papers report the experience with genetic counselling and testing in at-risk relatives for AD and FTD [12–14].

In Italy, a network of research centres with expertise in hereditary dementia developed a consensus research protocol for genetic counselling and testing of affected individuals and healthy at-risk relatives with familial AD and FTD within the Italian Dominantly Inherited Alzheimer’s and Frontotemporal Network (IT-DIAfN) project [15]. The IT-DIAfN protocol has been implemented within a research environment in centres participating into the IT-DIAfN project.

The aim of this study was to evaluate feasibility and acceptability of the genetic counselling and testing process for familial dementia, as undertaken according to the IT-DIAfN protocol. We appraised the 4 years’ experience in one IT-DIAfN research centre to determine whether the procedures set out in each phase of the protocol can be implemented in a research setting and offered to both affected individuals and at-risk relatives; this appraisal may provide a ground of evidence for future improvements.

## Methods

### Protocol

From January 2015 to March 2019, the IT-DIAfN protocol was implemented at the IRCCS Istituto Centro San Giovanni di Dio Fatebenefratelli, Brescia, Italy, by a multidisciplinary team including a geneticist (EDM), a geriatrician (SG) and a psychologist (AM). The protocol was approved by local ethical committee. All the affected individuals (or legal representatives) and at-risk relatives signed two informed consent forms, one for genetic counselling and one for genetic testing. All the clinical and genetic data were managed in accordance with the General Data Protection Regulation (UE n. 679/2016) and the “Ethical code for the use of biological material for research or experimental purpose” elaborated by the local Ethics Committee. Genetic counselling was conducted according to the core ethical principles of autonomy and non-directiveness.

The IT-DIAfN protocol was described in detail elsewhere [15]. In brief, inclusion criteria were as follows: (i) affected individuals with a family history suggestive of familial AD or FTD and (ii) healthy at-risk relatives of affected individuals with proven autosomal dominant mutation for AD or FTD. Each individual has to be ≥ 18 years old. Prenatal counselling and diagnosis were not included in the protocol. A suggestive family history was defined based on the presence of (i) at least three affected first-degree relatives in two generations, irrespectively of the age at onset, or (ii) at least two affected first-degree relatives in two generations, with at least one with onset at ≤ 65 years, or (iii) one affected family member with onset at ≤ 60 years or with a suggestive clinical phenotype (e.g. dementia with atypical presentation, recurring presence in other relatives, peculiar geographic origin).

The IT-DIAfN protocol consisted of (i) pre-test consultation, (ii) genetic testing, (iii) genetic test result disclosure and (iv) follow-up. Participants were allowed to withdraw from the protocol at any stage and could be readmitted by starting again with pre-test consultation.

Pre-test consultation comprised at least one counselling session for affected individuals and at least two counselling sessions for at-risk relatives. Additional sessions can be scheduled, if deemed appropriate by the team or requested by participants. For at-risk relatives, the participation of a supporting person was highly recommended, but not mandatory. During sessions, participants received information about familial forms of dementia and details of the protocol (number of sessions, disclosure, follow-up). Supportive information was given regarding the genetic risk in the family and its clinical, psychological, social, and ethical implications, together with the impact on family members. Possible influence of family members in the decision to undergo genetic testing was explored during the counselling sessions. The implications of both positive and negative genetic test results were discussed. Reasons for testing were discussed and any questions or doubts were addressed.

A psychological assessment was administered, including scales of personality traits (Big Five Questionnaire, [16]), anxious and depressive symptoms with evaluation of past or present suicidal thoughts or attitudes (Beck Depression Inventory, [17]; Hamilton Depression Rating Scale, [18]; State-Trait Anxiety Inventory, [19]), quality of life (12-Item Short Form Health Survey, [20]; World Health Organization Quality of Life-short version, [21]), coping strategies (Brief COPE, [22]), resilience (Resilience Scale for Adults, [23]) and health-related beliefs (Multidimensional Health Locus of Control scale, [24]). The severity of dementia was evaluated with the Clinical Dementia Rating (CDR) scale [25]. All these scales were re-administered during the follow-up visits, except for the Big Five Questionnaire and the CDR scale.

Before blood sample collection and genetic testing, the team discussed the outcome of the counselling phase. A supportive discussion was conducted with the participant to assess the comprehension of information received, and remaining doubts or questions were addressed. The results of psychological assessment were disclosed and discussed with the participant.

After genetic testing, disclosure of the result was done with in-person verbal and written communication.

Follow-up consisted of three counselling sessions, at 1, 6 and 12 months from disclosure. Psychological impact of both positive and negative genetic test results on personal, interpersonal and familial dynamics was assessed and psychological scales were re-administered.

The IT-DIAfN research project about genetic counselling and testing for inherited dementia was disseminated through the website of the IT-DIAfN project [26] and of our Institution [27].

### DNA analysis

Blood samples were taken at the IRCCS Istituto Centro San Giovanni di Dio Fatebenefratelli or at home for affected individuals who cannot be transported outside or were institutionalized.

At its inception, the IT-DIAfN protocol had developed a decision tree to assist in the search of causative mutation in affected individuals [15]. Since 2016, the mutation search was performed by the mean of next-generation sequencing (NGS) technology [28]. As a consequence, the prioritization procedure was no longer adopted.

Through the NGS technology, a wider panel of genes were analysed: *APP*, *PSEN1*, *PSEN2*, *GRN*, *MAPT*, prion protein (*PRNP*), charged multivesicular body protein 2B (*CHMP2B*), fused in sarcoma (*FUS*), TAR DNA-binding protein (*TARDBP*), valosin-containing protein (*VCP*), triggering receptor expressed on myeloid cells 2 (*TREM2*). From 2016 to 2017, the NGS analysis was performed at the IRCCS Istituto Centro San Giovanni di Dio Fatebenefratelli, according to standard procedures (Illumina MiSEq platform), or through the use of the Ion Torrent Personal Genome Machine (PGM) (Thermo Fisher Scientific, Waltham, MA USA) sequencer as NGS platform. Details were reported elsewhere [29]. Since 2018, the NGS technology was applied at the IRCCS Istituto Neurologico Carlo Besta of Milan, by the mean of the Nextera Flex for Enrichment-IDT protocol and the Illumina MiSeq platform, according to the manufacturers’ recommendations. C9orf72 analysis was performed by polymerase chain reaction (PCR) and size estimation of the amplified fragments.

## Results

Seventy-six families requested genetic counselling. The main reason was the concerns of either a spouse or a first-degree relative of an affected individual about a possible genetic aetiology of the disease suggested by an early onset of dementia or by the presence of more than a family member affected by dementia. About 50% of the families were excluded because the diagnosis was dementia other than AD or FTD (i.e. vascular dementia or dementia of uncertain aetiology) and/or the proband was deceased. Twenty affected individuals from 17 of these families satisfied the family history criteria of the IT-DIAfN protocol for suspected inherited dementia (17 for AD, 2 for FTD, 1 for inclusion body myopathy with Paget disease of bone and frontotemporal dementia, IBMPFD) and were included. The affected individual with suspected IBMPFD was not symptomatic for FTD or dementia, but for Paget disease of the bone and was included considering the family history highly suggestive for IBMPFD. Three additional families with a previously ascertained inherited dementia were also included.

### Affected individuals

Table 1 shows that affected individuals had a mean age of 66.0 + 12.8 years [range 34–81], were more frequently female (90%) and came from the Northern of Italy (Lombardy, Piedmont, Veneto, Trentino Alto Adige, Emilia Romagna), except for two families, one from Central Italy (Umbria) and one from Southern Italy (Puglia). Suspected diagnosis was inherited AD in 17 patients (85%), inherited FTD in 2 (10%) and IBMPFD in 1 (5%). Mean age at dementia onset was 59.1 + 10.0 years [range 33–70]. The majority of affected individuals had moderate-severe cognitive impairment (CDR score > 2 in 79% of patients).

**Table 1:** Sociodemographic and clinical features of 31 participants included in the IT-DIAfN protocol

	Affected individuals (*n* = 20)	Healthy at-risk relatives (*n* = 11)
*Sociodemographics*		
Age (years)	66.0 + 12.8	38.4 + 11.7
Gender (F)	18 (90%)	8 (73%)
Education		
Middle school	14 (70%)	2 (18%)
High school	3 (15%)	6 (55%)
University	3 (15%)	3 (27%)
Married	19 (95%)	4 (36%)
*Geographical origin*		
Northern Italy	18 (90%)	11 (100%)
Centre of Italy	1 (5%)	0 (0%)
Southern Italy	1 (5%)	0 (0%)
*Suspected diagnosis*		
Inherited AD	17 (85%)	–
Inherited FTD	2 (10%)	–
IBMPFD	1 (5%)	–
*Age at dementia onset* (years)	59.1 + 10.0	–
*Cognitive severity*		
Clinical Dementia Rating scale score		
0	1 (5%)	–
0.5–1	5 (25%)	–
≥ 2	14 (70%)	–
*Expected time to disease onset* (years)*		
0–15	–	4 (36%)
16–30	–	6 (55%)
> 30	–	1 (9%)

*Computed relative to mean disease onset in the family

*AD* Alzheimer’s disease, *FTD* frontotemporal dementia, *IBMPFD* inclusion body myopathy with Paget disease of bone and frontotemporal dementia

Eleven affected individuals (8 families) had a legal representative who attended genetic counselling. Fourteen patients (11 families, 65%) did not undergo pre-test psychological assessment due to severe cognitive impairment.

**Fig. 1:**
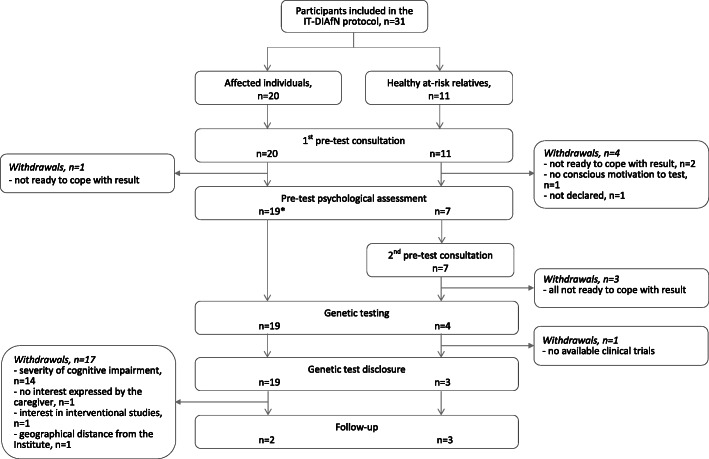
Flow of the genetic counselling and testing process. *Not applicable in 14 patients due to severe cognitive impairment

Figure 1 shows the flow of the genetic counselling and testing process. Two affected individuals completed the entire protocol, including the follow-up. Among the 18 affected individuals who did not complete the whole protocol, one left after the pre-test consultation, due to worries about the potential negative impact of a positive test result. Seventeen affected individuals did not undergo follow-up after the genetic test disclosure, 14 (82%) due to severe cognitive impairment and 3 for the following reasons: geographical distance from the Institute; no interest in completing the follow-up expressed by the caregiver; willingness to search for interventional research studies (*n* = 1, 6%, respectively).

For the two affected individuals (one carrier without cognitive impairment and one non-carrier) who completed the follow-up at 12 months from the disclosure of the genetic test result, no catastrophic reactions, such as a major depressive disorder, psychiatric hospitalization or suicide attempt, were reported to date. A pathogenic mutation was found in 6 affected individuals (Table 2).

**Table 2:** Pathogenic mutations identified in 6 affected individuals (6 families)

Family code	Diagnosis	Gene	Mutation	
			(nucleotide change)	(amino acid change)
30_1	Inherited AD	*PSEN2*	NM_000447.3: c.717G>A	M239I^a^
36_1	IBMPFD	*VCP*	NM_007126.5: c.277C>T	R93C^b^
L029	Inherited FTD	*GRN*	NM_002087.3: c.813_816del	Thr272fs^c^
L031	Inherited AD	*PSEN2*	NM_000447.3: c.717G>A	M239I^a^
L035	Inherited FTD	*MAPT*	NM_005910.5: c.915+3G>A	IVS10+3G>A^d^
L036	Inherited AD	*PSEN1*	NM_000021.4: c.1129A>T	R377W^e^

*AD* Alzheimer’s disease, *FTD* frontotemporal dementia, *IBMPFD* inclusion body myopathy with Paget disease of bone and frontotemporal dementia, *PSEN* presenilin, *VCP* valosin-containing protein, *GRN* granulin, *PSEN2* presenilin2, *MAPT* microtubule-associated protein tau

^a^rs63749884 (NC_000001.11:g.226888979G>A)

^b^rs1554669087 (NC_000009.11; g.35067913C>T)

^c^rs63749877 (NC_000017.11:g.44351141_44351144delCACT)

^d^rs63750013 (NG_007398.1: g.120985G>A)

^e^rs1555357544 (NC_000014.9:g.73211942A>T)

**Fig. 2:**
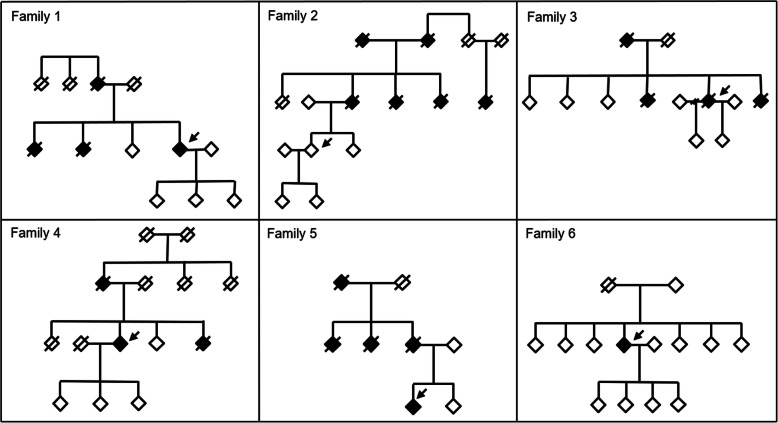
Pedigree of 6 families with a pathogenic mutation identified within the IT-DIAfN protocol. Age and gender were not reported to preserve anonymity; arrows indicate probands

The clinical features of index cases are reported below. Pedigrees are depicted in Fig. 2.

#### Family 1

The pedigree consisted of four affected first-degree relatives in two generations; for three of them, including the index case, the diagnosis was AD, while for the other affected individual the diagnosis was dementia of undetermined aetiology. Considering the three affected individuals for which the age at onset was known, the mean age of symptoms onset was 57 ± 3 years (range 54–60). The mean age of death of affected individuals was 65 ± 9 years. The index case was a 58-year-old female, who was married and had three children. She was from the Northern Italy. Physical health was good, reporting only glaucoma. At 54 years, she had feelings of sadness, anxiety and an episode of visual hallucinations. A diagnosis of anxious-depressive disorder was made and a therapy with serotoninergic antidepressants was started. At the same time, she showed progressive memory impairment and an episode of spatial disorientation. At 56 years, she underwent a cognitive evaluation, showing mild cognitive deficit (the MMSE test was 22/30). A cerebrospinal fluid exam showed decreased Abeta42 and increased tau levels, and a diagnosis of AD was made. Therapy with acetylcholinesterase inhibitors was started. At 57 years, the MMSE score was 20/30. At 58 years, the patient was included in the IT-DIAfN protocol, and the Met239Ile mutation was identified in *PSEN2* gene.

#### Family 2

The pedigree consisted of six affected first-degree relatives in three generations. Two had a diagnosis of FTD and Paget disease of the bone, one of AD and Paget disease of the bone, one of Parkinson disease and Paget disease of the bone and one of AD. The index case had a diagnosis of Paget disease of the bone. Considering the three affected individuals with dementia for which the age at onset was known, the mean age of dementia onset was 66 ± 7 years (range 61–77). The mean age of death was 73 ± 7 years. The index case was a 47-year-old male, who was married and had two children. He was from Central Italy. At 44 years, the patient suffered symptoms of coxofemoral osteoarthritis, treated with zoledronic acid and methylprednisolone. A diagnosis of Paget disease of the bone in the left pelvis was made. Bone scintigraphy confirmed the presence of Paget disease of the bone, with multifocal localization. This patient had no any cognitive deficits. At 47 years, the patient requested genetic counselling due to concerns about a possible genetic aetiology of the disease and was included in the IT-DIAfN protocol. The R93C mutation was identified in *VCP* gene.

#### Family 3

The pedigree consisted of four affected first-degree relatives in two generations; for three of them, the diagnosis was dementia of undetermined aetiology, while for the index case the diagnosis was FTD. Considering the two family members for which the age at onset was known, the mean age of symptom onset was 61 ± 1.4 years (range 60–62). The mean age of death of affected individuals was 62.3 ± 6.7 years. The index case was a 63-year-old female, married with two children. She was from the Northern Italy. No physical comorbidity was reported. At 62 years old, she showed progressive apathy, language difficulties with reduced speech, and compulsive behaviour associated to cigarette smoking and food intake. At 63 years, a neuropsychological evaluation demonstrated moderate cognitive impairment with language comprehension and production deficit. A FDG-PET showed marked frontal hypometabolism, left more than right. A diagnosis of frontotemporal dementia-behavioural variant was made. At 63 years, the patient was included in the IT-DIAfN protocol, and the Thr272fs mutation was identified in *GRN* gene.

#### Family 4

The pedigree consisted of three affected first-degree relatives in two generations: two affected individuals, including the index case, received a diagnosis of AD while for the other the diagnosis was dementia of undetermined aetiology. The mean age at onset was 64.6 ± 2.3 years (range 62–66). The mean age of death was 72 ± 4.2 years. The index case was a 76-year-old female, widow and with three children. She was from the Northern Italy. Physical comorbidity included glaucoma with consequent low vision and hypothyroidism under medical treatment. At 64 years, the patient showed progressive memory and attention deficits and spatial disorientation. At 66 years, a neuropsychological evaluation showed deficits on verbal and non-verbal memory, attention, verbal production and visuospatial abilities (the MMSE score was 19/30). A FDG-PET evidenced bilateral temporo-parietal and precuneus hypometabolism. A clinical diagnosis of AD was made. At 76 years, the patient was included in the IT-DIAfN protocol, when cognition was severely impaired. The Met239Ile mutation was identified in *PSEN2* gene.

#### Family 5

This pedigree consisted of five affected first-degree relatives in two generations: the index case received a diagnosis of FTD, for the others, the diagnosis was dementia of undetermined aetiology. Considering the two affected individuals for which the age at onset was known, the mean age of symptom onset was 40 ± 9.9 years (range 33–47). One of these two patients died at 56 years old. The index case was a 35-year-old male, who was married. He was from the Northern Italy. No physical comorbidity was reported. At 32 years, the patient showed mild and progressive impairment on memory (difficulties in recall recent events and even very salient events from own past life) and language (difficulties in identifying the meaning of words), and behavioural disturbances (irritability, absence of libido, affective flattening, poor personal hygiene care, excessive intake of sweets). At 34 years, a neuropsychological evaluation showed deficit in verbal memory and language (denomination and semantic fluency). A cerebrospinal exam was negative for AD. FDG-PET showed bilateral hypometabolism in temporal poles and mesial temporal lobe, more evident on the left side. A diagnosis of frontotemporal neurodegeneration was made. At 35 years the patient was included in the IT-DIAfN protocol, and the IVS10+3G>A genetic mutation was identified in *MAPT* gene.

#### Family 6

The index case was a 53-year-old female, with four children. She is the only affected individual in the family. She was from the Northern Italy. No relevant comorbidity was reported. The patient suffered from post-partum depression in each pregnancy. At 52 years, she had an episode of paranoid disorder, treated with antipsychotics. One year before, the patient reported progressive episodic memory impairment and anomia, with slight functional impairment (difficulties in organizing her work). She had partial insight. At 53 years, a neuropsychological evaluation showed deficits in long- and short-term verbal memory, attention and executive functions (the MMSE score was 25/30). Left temporal hypometabolism was demonstrated by FDG-PET, while flutemetamol-PET showed the presence of cortical amyloid deposition. A diagnosis of AD was made. At 53 years, the patient was included in the IT-DIAfN protocol, and the Arg377Trp mutation was identified in *PSEN1* gene.

### Healthy at-risk relatives

We had 9 families with an identified pathogenic mutation whose at-risk relatives were potentially eligible for predictive testing. Of these 9 pathogenic mutations, six were identified within the IT-DIAfN protocol and three elsewhere (*GRN* T272fs; *PSEN1* E184G; *PSEN1* L392V). Thirty-eight members of these 9 families were at 50% risk for the pathogenic mutation. Sixteen were siblings, 19 adult child, and 3 children under 18 years of age. Eleven out of the 38 at-risk relatives asked for genetic testing and were included in the protocol. The most of these (8/11) were the at-risk relatives of affected individuals enrolled in the IT-DIAfN protocol.

Table 1 shows that the average age of at-risk relatives was 38.4 + 11.7 years [range 19–53]; they were more frequently female (73%) and highly educated (82% had high school graduation or more); 4 (36%) were married. Time expected to disease onset was less than 15 years in about one third of the subjects. The main reasons to ask for a predictive genetic test were the expectation to have access to experimental drugs (73%), to support research (64%) and to plan the future (64%). Thirty-six percent of the subjects wanted to make reproductive choices, 27% wanted to reduce uncertainty about their own genetic status and 27% to have time for family planning in the event of an unfavourable outcome. One subject (1%) did not have a clear awareness of the reasons to ask the genetic test.

Figure 1 shows the flow of the genetic counselling and testing process. Three out of 11 at-risk relatives completed the protocol (follow-up counselling sessions with psychological assessment are ongoing). The remaining eight subjects left the protocol during pre-test consultation, five of them (63%) due to a gained awareness of not being ready to cope with a potential unfavourable test result. One person left the protocol not having gained a conscious motivation to carry out the genetic test, and the last one without providing a reason. One subject did not ask for the genetic test result after genetic testing, because at that moment no clinical trials were available.

For the three subjects (one carrier and two non-carriers belonging to the same family), who are completing the follow-up, no catastrophic reactions were reported to date.

## Discussion

This study describes the experience of a research centre with specific expertise in dementia with the IT-DIAfN protocol, a structured genetic counselling and testing protocol for familial AD and FTD. The IT-DIAfN protocol was implemented in both affected individuals and healthy at-risk family members, according to the current guidelines of the American College of Medical Genetics and the National Society of Genetic Counsellors [10], which state that genetic testing for AD should only occur in the context of genetic counselling, for both affected individuals and at-risk relatives.

The current guidelines, in turn, were mainly based on the large body of evidence produced during the decades of experience with HD, making the HD genetic counselling and testing protocol the gold standard for late-onset neurodegenerative diseases. However, clinical and genetic differences between HD and dementia (prominent movement disorder usually not associated with early dementia and single-gene mutation in HD), the expansion of genetic tests for dementia and the availability of clinical trials for prevention and treatment, raised new issues requiring specific guidelines and protocols for dementia [30].

Experiences of genetic counselling and testing protocol implementation in affected individuals were not reported in the literature. In the genetic counselling program for dementia in Spain [11], a genetic study was offered to 87 patients with suspected familial AD and FTD or genetically determined prion diseases, directly after obtaining a written consent. A specific counselling protocol was applied only to at-risk relatives [11]. The reasons for genetic testing in affected individuals were not reported.

In our experience, the genetic testing for affected individuals was mainly requested by either a spouse or a first-degree relative who was concerned about the occurrence of a possible familial form of dementia. Only two patients with early-onset dementia (< 50 years) were included in the IT-DIAfN protocol upon clinical indication. In common practice, in fact, genetic testing in dementia is requested for affected individuals with a family history strongly indicative of early-onset autosomal dominant dementia, in whom the identification of a mutation may result in definitive diagnosis. This request was done within a clinical diagnostic work-up, and the patient unlikely had access to a research program including genetic counselling. Nonetheless, the increased awareness in genetics of dementias increased requests for information. The public dissemination of the IT-DIAfN research project of genetic counselling and testing allowed the individuals concerned about own genetic risk to ask for the genetic test. Of note, in a previous study on FTD, we revealed that a high proportion of patients belonging to high-risk pedigrees asked for genetic counselling; requests decreased according to the estimated family risk [31].

In the present series, one affected individual left the IT-DIAfN protocol after the pre-test consultation, because of the turned awareness of not being ready to cope with a potential unfavourable result. This occurrence underlines that, in the context of genetic counselling, the uptake of genetic testing should not be considered the final aim. When genetic testing of an affected individual was requested by a relative, genetic counselling was critical to explore attitudes towards pursuing genetic testing [10], the potential impact on the family and possible lack of interest of the patient to know the result of the genetic test. In this context, the multidisciplinary counselling team ensured a comprehensive care of the entire family including affected individuals, caregivers and at-risk relatives. According to the most acknowledged definition of genetic counselling [32], all family members involved were offered an open communication process, structured to allow the comprehension of medical and genetic facts, their implications and the meaning of each available option, with the aim of the best adjustment with the illness or the risk status.

The high rate of lost-to-follow-up suggested that a revision of the IT-DIAfN protocol for affected individuals would be considered. The lack of psychological assessments—most are self-administered scales—is not astonishing. Psychological assessment included in the IT-DIAfN protocol was developed in order to have a comprehensive evaluation of emotional and psychological conditions of the participant, and monitor the impact of the test result on the personal, family and social balance of the person. At the time of the protocol development, we had not included an evaluation of psychological state of mind of caregiver. However, patient and caregiver represent a dyad with a synergistic relationship and mutual psychological influence [33]. The active participation of the caregiver in assessing and reviewing the psychological impact of the genetic test procedure could facilitate the involvement of the family and allow to reveal mutual psychological influences within the patient-caregiver dyad. Should the follow-up visits be perceived too demanding, remote meetings could be offered to families in which geographical distance from the research centre limits in-person follow-up visits, as suggested by preliminary evidence [34].

We identified 5 genetic mutations in 6 families. These mutations had been reported as “pathogenic” in the dedicated mutation database for AD and FTD [35]. We already reported the genetic mutations identified in families 1 thru 4 elsewhere [29]. In families 1 and 4, the Met239Ile mutation was identified in the *PSEN2* gene. It was first reported in an Italian family [36] and then in two additional Italian families [37, 38]. No common ancestor has been established, and a specific search was beyond the scope of this study. In the family 2, the R93C mutation was identified in the *VCP* gene. It was first reported in a family of unknown ancestry [39], then in a German family [40], in a family of unknown ancestry [41], and in a Brazilian family [42]. In the family 3, the Thr272fs mutation was identified in *GRN* gene. It was first reported in an Italian family [43] and in other 34 Italian and one French families [44–49]. In the family 5, the IVS10+3G>A genetic mutation was identified in the *MAPT* gene. It was described in three families of European ancestry [50–52], one American family [53] and one Japanese family [54]. In the family 6, the Arg377Trp mutation was identified in *PSEN1* gene. It was first reported in a French family [55] and then in an Italian one [56]. In these two families, as well as in the one described in the present study, family history was unremarkable. Overall, no distinctive clinical phenotypes were evident in our index cases relative to the literature. Only slight differences in age at onset were noted in the index cases of families 4 and 5 relative to the previously reported age ranges (64 vs 44 to 58 years, and 33 vs 38 to 59 years, respectively).

In healthy at-risk relatives, the uptake rate of predictive testing (i.e. the number of predictive tests performed as a proportion of the 50% at-risk members of families with an identified pathogenic mutation) was 8% (3 out of 38). Our result was consistent with the uptake of 8% reported by the experience of the University of Washington in persons at-risk for AD and FTD [13] and slightly higher to that (3%) reported in the experience of genetic counselling for FTD in the Netherlands [14]. A direct comparison with the literature data in HD was limited by methodological differences in the approaches used to calculate the uptake rate [57]. The figures varied between 5 and 45%, even if an uptake ratio lower than 20% were more consistently reported [57, 58]. The pre-familial amyotrophic lateral sclerosis (FALS) study, a prospective, observational study of individuals at risk of FALS, reported a very high uptake rate (70%). However, the design of the study can explain this discrepancy [59]. Furthermore, our figure of 8% may be underestimated because about 40% of the 50% at-risk members were unable to access predictive testing being less than 18 years old or were not aware of the presence of a pathogenic mutation in the family. To preserve confidentiality, after withdrawal we refrained from exploring whether and how the genetic information was shared within the family.

As regards the sociodemographics of at-risk relatives included in the protocol, the gender ratio (73% women) was consistent with a previous study of predictive genetic counselling for AD, in which 60% of at-risk participants were women [13] and with the literature of predictive testing for HD, reporting a female predominance among those tested [57, 58]. Also the mean age close to 40 years in our series was in line with the literature of predictive testing for AD [13], inherited dementias [60] and HD [58, 61]. The prevalence of highly educated subjects (82% with high school o graduation) in our series was higher than previously reported in both dementia and HD [13, 58, 60, 62]. Notably, in our study, the 3 at-risk relatives with university degree were the only ones who completed the protocol, suggesting that high education may be one of the variables associated to greater ability to deal with the process of genetic counselling. This finding would encourage our community to take into account the unequal access to the advanced health technologies, such as molecular genetic analyses, and proactively act to reduce it. Gender can also be a variable related to higher attitude of women to cope with an unfavourable result [63] or to seek medical attention [64]. For 4 out of 11 at-risk relatives (36%), the expected time to disease onset was less than 15 years. Interestingly, 3 out of them were those who completed the entire protocol, suggesting that proximity to the expected time to disease onset may be a factor related to the willingness to know own genetic status.

The most common reasons for requesting genetic testing, expressed by more than 60% of at-risk relatives, were the opportunity to participate in future clinical trials of experimental drugs, to support research and to plan the future. Considering the literature on reasons for predictive testing in HD, the most common reported (> 70%) were different, i.e. to reduce uncertainty about the risk of illness, reproductive decision-making and future planning [57, 58]. The hope for future treatment, when cited, was the reason in less than 10% of cases [57]. In an observational study of 24 subjects at risk for FTD or AD, the main reasons (> 75%) for requesting genetic testing were financial and family planning, and relief from worry and anxiety [13]. Possible eligibility for future treatment trial was the less common cited reason [13]. The recent availability of clinical trials of experimental drugs for inherited AD [65] can explain this change of demand for genetic testing. High prevalence of motivation related to support research can be also explained by awareness that our Institution was a research centre and that the IT-DIAfN protocol was developed in the context of a research project. This view was supported by a study on a research protocol developed to evaluate the impact of genetic susceptibility testing among asymptomatic relatives of people with AD [66]. The most common reasons (> 85%) for seeking testing were comparable with our findings, i.e. to contribute to AD research, to hope that effective treatments will be developed and to arrange personal affairs [66].

The rate of withdrawals from the protocol of our at-risk relatives was 72%. The figure was higher than that reported in studies of predictive testing for HD, showing a withdrawal rate between 12 and 51% [58]. In the experience with genetic counselling for dementia in Spain, 30 out of 54 (56%) at-risk subjects who entered genetic counselling protocol decided to not receive the genetic test result [11]. In the experience with genetic counselling for FTD in the Netherlands, the rate of withdrawal was very similar (7/13, 54%) [14], while in the Washington University series 1 out of 15 at-risk subjects (7%) did not proceed with the genetic testing [13]. Some peculiar characteristics of participants, such as emotional vulnerability due to young age—in the present series two participants were 19 and 24 years old, can partially explain the different rate of withdrawals. However, the limited number of enrolled individuals and the heterogeneous setting across studies do not allow to infer definite explanations from observed frequencies.

The most common reason for leaving the protocol in the present study was the same reported in the literature for HD, that is worry of being unable to cope with an unfavourable result [58, 67]. The analyses of data from multiple cohorts of families with HD and other neurodegenerative diseases demonstrated that self-selection occurs. In most cases, the reason to choose not to proceed with the test is driven by the concern about the possible unfavourable result—worry, anxiety, presumed inability to cope with an unfavourable result are all possible reasons to choose to not to know. Conversely, individuals who solicit testing have stronger coping abilities and are more able to handle stressful situations [68]. In the context of an extended counselling protocol, a high rate of self-withdrawal could be considered as a favourable outcome. The time lapse between the pre-test consultations and the final decision to undergo the test had a major role in providing a proper decision-making process and, for the counselling team, to evaluate the counselee’s emotional stability.

In a minority of cases, the participants left the protocol because of the absence of a conscious motivation to undergo the genetic test, or without providing a reason. We observed that emotional stability and specific motivation had great relevance in determining the decision of knowing own genetic status. A study in HD [69] found that the motivation to testing had a strong predictive value in foreseeing personal reactions to the disclosure of the genetic status, independently from ego-strength and test result. In general, the literature in HD showed that those who opt for genetic testing were the best equipped emotionally to deal with the test results [70].

The current unavailability of clinical trials of experimental drugs was the reason of withdrawals in one of our at-risk relatives after genetic testing. As underlined above, the research setting in which genetic counselling had been provided possibly induced the expectation of access to experimental drugs. To this regard, genetic counselling was critical to deeply investigate if the counselee understood all the possible implications associated to the disclosure of his/her genetic status. The access to clinical trials was discussed with the counselees and the families during counselling sessions as a possible option, which could be offered to selected individuals in the future.

At present, no catastrophic reactions, defined as major depressive disorder, psychiatric hospitalization or suicide attempt occurred. For the three at-risk siblings who completed the protocol, the familial relationship strengthened the mutual support in the decision-making process and helped the individuals to perceive their ability to deal with an unfavourable outcome. This finding is consistent with previous studies [11–14, 60]. Long-term follow-up studies are warranted to investigate the personal impact of the test result, as well as the social consequences of an unfavourable outcome such as discrimination or stigma, and the impact of sharing the genetic information within the family.

All families informally expressed satisfaction of having pursued the genetic counselling and testing, in line with previous literature in HD [58]. However, a formal assessment of satisfaction had not been implemented. We believe that a structured examination of families’ satisfaction should be included as an additional tool to evaluate the outcomes of the counselling and testing procedure.

The workflow of molecular analyses originally developed has been adapted to our centre as far as concerns the decision tree for mutation analysis, according to the innovations that could be easily foreseen at that time [15]. The massive sequencing approach based on NGS platforms has become the first tier for molecular diagnosis of heterogeneous genetic disorders such as AD and FTD, integrated with PCR analyses of the *C9orf72* hexanucleotide repeat expansion and the *PRNP* octapeptide repeat region. In a research setting, massive sequencing may be an added value. In a recent study, we demonstrated that the polygenic risk score may contribute to interpret the role for the genetic load in families without single-gene mutations associated to autosomal dominant forms of dementia [29].

Based on this evaluation, future changes may improve the IT-DIAfN protocol. A psychometric assessment of the state of mind of caregivers should be implemented [10], as well as a formal measurement of participants’ satisfaction. In view of translation in routine clinical practice, we acknowledge that the entire procedure is demanding, especially in terms of human resources. A few steps of the protocol that are currently held in-person may be replaced by remote consultations, provided that a dedicated platform is securely implemented. It is apparent that the availability in the future of innovative drugs, or preventive strategies able to change the course of dementia, will lead the neurogenetics community to develop novel genetic counselling and testing procedures for familial cases.

### Limitations

The results of the present study should be considered preliminary because of the small number of participants and the relatively short follow-up. Genetic counselling and testing process in familial dementia can be improved on the basis of further evaluation of the IT-DIAfN protocol in larger cohorts and different settings. The limited number of affected individuals undergoing the psychological assessment prevented a full evaluation of the protocol effectiveness. Selection bias (i.e. inclusion of a high number of affected individuals with severe cognitive impairment) may be related to the research setting in which the IT-DIAfN protocol was implemented, limiting the referral of patients with mild dementia. Effectiveness and selection bias of at-risk relatives pursuing the genetic testing (i.e. personality or psychological characteristics that promoted self-selection of individuals who are able to deal with the counselling process and test result) deserves to be further explored (manuscript in preparation).

## Conclusions

This study showed that the structured procedures of genetic counselling and testing, with planned pre- and post-test counselling sessions, can be implemented in both affected individuals and at-risk relatives in a research setting. The development of an optimized protocol is an essential prerequisite for a safe and effective integration of genetic counselling and testing for dementia in the health care system.

## Data Availability

The data which support this study are not publicly available, but may be provided upon reasonable request.

## Abbreviations

IT-DIAfN: Italian Dominantly Inherited Alzheimer’s and Frontotemporal Network
AD: Alzheimer’s disease
FTD: Frontotemporal dementia
HD: Huntington’s disease
IBMPFD: Inclusion body myopathy with Paget disease of bone and frontotemporal dementia
APP: Amyloid precursor protein
PSEN: Presenilin
GRN: Granulin precursor
MAPT: Microtubule-associated protein tau
C9orf72: Chromosome 9 open reading frame 72
VCP: Valosin-containing protein
CDR: Clinical Dementia Rating
NGS: Next-generation sequencing technology
PRNP: Prion protein
CHMP2B: Charged multivesicular body protein 2B
FUS: Fused in sarcoma
TARDBP: TAR DNA-binding protein
TREM2: Triggering receptor expressed on myeloid cells 2
PGM: Personal Genome Machine
PCR: Polymerase chain reaction
MMSE: Mini-Mental State Examination
Abeta42: Beta-amyloid peptide 42
FDG-PET: Fluorodeoxyglucose-positron emission tomography
FALS: Familial amyotrophic lateral sclerosis

## References

[1] World Health Organization. Factsheet, Dementia. 2012. https://www.who.int/news-room/fact-sheets/detail/dementia. Last update 19 September 2019. Accessed 4 June 2020.

[2] Onyike CU, Diehl-Schmid J (2013). The epidemiology of frontotemporal dementia. Int Rev Psychiatry.

[3] Bateman RJ, Aisen PS, De Strooper B, Fox NC, Lemere CA, Ringman JM (2011). Autosomal-dominant Alzheimer’s disease: a review and proposal for the prevention of Alzheimer’s disease. Alzheimers Res Ther.

[4] Rohrer JF, Guerreiro R, Vandrovcova J, Uhphill J, Reiman D, Beck J (2009). The heritability and genetics of frontotemporal lobar degeneration. Neurology..

[5] Greaves CV, Rohrer JD (2019). An update on genetic frontotemporal dementia. J Neurol.

[6] Goldman JS (2015). Genetic testing and counselling in the diagnosis and management of young-onset dementias. Psychiatr Clin North Am.

[7] Kayson E, Eberly S, Anderson KE, Marder K, Shoulson I, Oakes D (2019). Huntington study group PHAROS investigators. The prospective Huntington at-risk observational study (PHAROS): the emotional well-being, safety and feasibility of long-term research participation. J Huntingtons Dis.

[8] Pitini E, D’Andrea E, De Vito C, Rosso A, Unim B, Marzuillo C, Federici A, Di Maria E, Villari P (2019). A proposal of a new evaluation framework towards implementation of genetic tests. PLoS One.

[9] Pitini E, De Vito C, Marzuillo C, D’Andrea E, Rosso A, Federici A, Di Maria E, Villari P (2018). How is genetic testing evaluated? A systematic review of the literature. Eur J Hum Genet.

[10] Goldman JS, Hahn SE, Catania JW, LaRusse-Eckert S, Butson MB, Rumbaugh M (2011). American College of Medical Genetics and the National Society of Genetic Counselors. Genetic counselling and testing for Alzheimer disease: joint practice guidelines of the American College of Medical Genetics and the National Society of Genetic Counselors. Genet Med.

[11] Fortea J, Lladó A, Clarimón J, Lleó A, Oliva R, Peri J (2011). PICOGEN: five years experience with a genetic counselling program for dementia. Neurologia..

[12] Lannfelt L, Axelman K, Lilius L, Basun H (1995). Genetic Counselling in a Swedish Alzheimer family with amyloid precursor protein mutation. Am J Hum Genet.

[13] Steinbart EJ, Smith CO, Poorkaj P, Bird TD (2001). Impact of DNA testing for early-onset familial Alzheimer disease and frontotemporal dementia. Arch Neurol.

[14] Riedijk SR, Niermeijer MF, Dooijes D, Tibben A (2009). A decade of genetic counselling in frontotemporal dementia affected families: few counselling requests and much familial opposition to testing. J Genet Couns.

[15] Bocchetta M, Mega A, Bernardi L, Di Maria E, Benussi L, Binetti G (2016). Genetic counselling and testing for Alzheimer’s disease and frontotemporal lobar degeneration: an Italian consensus protocol. J Alzheimers Dis.

[16] Caprara GV, Barbaranelli C, Borgogni L. Big Five Questionnaire. Firenze: O.S. Organizzazioni Speciali; 1993.

[17] Beck AT, Ward CH, Mendelson M, Mock J, Erbaugh J. An inventory for measuring depression. Archives General Psychiatry 1961;4:561–571. 10.1001/archpsyc.1961.01710120031004 13688369

[18] Hamilton M (1960). A rating scale for depression. J Neurol Neurosurg Psychiatry.

[19] Spielberger CD (1989). State-trait anxiety inventory: bibliography.

[20] Apolone G, Mosconi P, Quattrociocchi L, Gianicolo EAL, Groth N, Ware JE Jr. Questionario sullo stato di salute SF-12 (Versione Italiana), Milano: Istituto di Ricerche Farmacologiche Mario Negri; 2005.

[21] WHOQOL group. Manuale per l’uso degli strumenti WHOQOL (Versione Italiana), Ginevra: Dipartimento di Salute Mentale, Organizzazione Mondiale della Sanità. Medicine. 1997;4:92–100.

[22] Carver CS (1997). You want to measure coping but your protocol’s too long: consider the brief COPE. Int J Behav Med.

[23] Friborg O, Barlaug D, Martinussen M, Rosenvinge JH, Hjemdal O (2005). Resilience in relation to personality and intelligence. Int J Methods Psychiatr Res.

[24] Wallston KA, Wallston BS, DeVellis R (1978). Development of the multidimensional health locus of control (MHLC) scales. Health Educ Monogr.

[25] Morris JC (1993). The clinical dementia rating (CDR): current version and scoring rules. Neurology..

[26] Italian DIAfN – Dominantly Inherited Alzheimer and Frontotemporal Network. http://www.gendem.it/en/home-en. Accessed 31 December 2016.

[27] Centro Alzheimer – web site of the Lab of Alzheimer’s Neuroimaging et Epidemiology. https://www.centroalzheimer.org/en/. Accessed 19 July 2020.

[28] Slatko BE, Gardner AF, Ausubel FM (2018). Overview of next-generation sequencing technologies. Curr Protoc Mol Biol.

[29] Bonvicini C, Scassellati C, Benussi L, Di Maria E, Maj C, Ciani M (2019). Next generation sequencing analysis in early onset dementia patients. J Alzheimers Dis.

[30] Goldman JS (2020). Predictive genetic counselling for neurodegenerative diseases: past, present, and future. Cold Spring Harb Perspect Med.

[31] Fostinelli S, Ciani M, Zanardini R, Zanetti O, Binetti G, Ghidoni R (2018). The heritability of frontotemporal lobar degeneration: validation of pedigree classification criteria in a northern Italy cohort. J Alzheimers Dis.

[32] Resta R, Biesecker BB, Bennett RL, Blum S, Hahn SE, Strecker MN (2006). National Society of Genetic Counselors’ Definition Task Force. A new definition of genetic counselling: National Society of Genetic Counselors’ Task Force report. J Genet Couns.

[33] Van’t Leven N, de Lange J, van der Ploeg ES, Pot AM (2018). Working mechanisms of dyadic, psychosocial, activating interventions for people with dementia and informal caregivers: a qualitative study. Clin Interv Aging.

[34] Hilgart JS, Hayward JA, Coles B, Iredale R (2012). Telegenetics: a systematic review of telemedicine in genetics services. Genet Med..

[35] Alzheimer Disease & Frontotemporal Dementia Mutation Database. VIB Uantwerp Center for Molecular Neurology, Belgium. https://uantwerpen.vib.be/mutations. Accessed 31 December 2018.

[36] Finckh U, Alberici A, Antoniazzi M, Benussi L, Fedi V, Giannini C (2000). Variable expression of familial Alzheimer disease associated with presenilin 2 mutation M239I. Neurology..

[37] Testi S, Fabrizi GM, Pompanin S, Cagnin A (2012). Autosomal dominant Alzheimer’s disease with early frontal lobe involvement associated with the Met239Ile mutation of presenilin 2 gene. J Alzheimers Dis.

[38] Tremolizzo L, Susani E, Mapelli C, Isella V, Bertola F, Ferrarese C, Appollonio I (2014). First report of PSEN2 mutation presenting as posterior cortical atrophy. Alzheimer Dis Assoc Disord.

[39] Guyant-Maréchal L, Laquerrière A, Duyckaerts C, Dumanchin C, Bou J, Dugny F (2006). Valosin-containing protein gene mutations: clinical and neuropathologic features. Neurology..

[40] Hübbers CU, Clemen CS, Kesper K, Böddrich A, Hofmann A, Kämäräinen O (2007). Pathological consequences of VCP mutations on human striated muscle. Brain..

[41] Krause S, Göhringer T, Walter MC, Schoser BGH, Reilich P, Linn J (2007). Brain imaging and neuropsychology in late-onset dementia due to a novel mutation (R93C) of valosin-containing protein. Clin Neuropathol.

[42] Fanganiello RD, Kimonis VE, Côrte CC, Nitrini R, Passos-Bueno MR (2011). A Brazilian family with hereditary inclusion body myopathy associated with Paget disease of bone and frontotemporal dementia. Braz J Med Biol Res.

[43] Benussi L, Binetti G, Sina E, Gigola L, Bettecken T, Meitinger T (2008). A novel deletion in progranulin gene is associated with FTDP-17 and CBS. Neurobiol Aging.

[44] Tremolizzo L, Gelosa G, Galbussera A, Isella V, Arosio C, Bertola F (2009). Higher than expected progranulin mutation rate in a case series of Italian FTLD patients. Alzheimer Dis Assoc Disord.

[45] Borroni B, Archetti S, Alberici A, Agosti C, Gennarelli M, Bigni B (2008). Progranulin genetic variations in frontotemporal lobar degeneration: evidence for low mutation frequency in an Italian clinical series. Neurogenetics..

[46] Carecchio M, Fenoglio C, De Riz M, Guidi I, Comi C, Cortini F (2009). Progranulin plasma levels as potential biomarker for the identification of GRN deletion carriers. A case with atypical onset as clinical amnestic mild cognitive impairment converted to Alzheimer’s disease. J Neurol Sci.

[47] Le Ber I, Camuzat A, Hannequin D, Pasquier F, Guedj E, Rovelet-Lecrux A (2008). Phenotype variability in progranulin mutation carriers: a clinical, neuropsychological, Imaging and Genetic Study. Brain.

[48] Benussi L, Ghidoni R, Pegoiani E, Moretti DV, Zanetti O, Binetti G (2009). Progranulin Leu271LeufsX10 is one of the most common FTLD and CBS associated mutations worldwide. Neurobiol Dis.

[49] Yu C-E, Bird TD, Bekris LM, Montine TJ, Leverenz JB, Leverenz E (2010). The spectrum of mutations in progranulin: a collaborative study screening 545 cases of neurodegeneration. Arch Neurol.

[50] Tolnay M, Spillantini MG, Rizzini C, Eccles D, Lowe J, Ellison D (2000). A new case of frontotemporal dementia and parkinsonism resulting from an intron 10+3-splice site mutation in the tau gene: clinical and pathological features. Neuropathol Appl Neurobiol.

[51] Neumann M, Mittelbronn M, Simon P, Vanmassenhove B, de Silva R, Lees A (2005). A new family with frontotemporal dementia with intronic 10+3 splice site mutation in the tau gene: neuropathology and molecular effects. Neuropathol Appl Neurobiol..

[52] Wierzba-Bobrowicz T, Lewandowska E, Zaremba J, Berdyński M, Żekanowski C, Stępień T (2014). Frontotemporal lobar degeneration with MAPT mutation in an Italian-Polish family. A case report. Folia Neuropathol.

[53] Spillantini MG, Bird TD, Ghetti B (1998). Frontotemporal dementia and parkinsonism linked to chromosome 17: a new group of tauopathies. Brain Pathol.

[54] Nan H, Takaki R, Shimozono K, Ichinose Y, Koh K, Takiyama Y (2019). Clinical and genetic study of the first Japanese FTDP-17 patient with a mutation of +3 in intron 10 in the MAPT gene. Intern Med.

[55] Wallon D, Rousseau S, Rovelet-Lecrux A, Quillard-Muraine M, Guyant-Maréchal L, Martinaud O (2012). The French series of autosomal dominant early onset Alzheimer’s disease cases: mutation Spectrum and cerebrospinal fluid biomarkers. J Alzheimers Dis.

[56] Borroni B, Pilotto A, Bonvicini C, Archetti S, Alberici A, Lupi A (2012). Atypical presentation of a novel presenilin 1 R377W mutation: sporadic, late-onset Alzheimer disease with epilepsy and frontotemporal atrophy. Neurol Sci.

[57] Baig SS, Strong M, Rosser E, Taverner NV, Glew R, Miedzybrodzka Z (2016). 22 years of predictive testing for Huntington’s disease: the experience of the UK Huntington’s Prediction Consortium. Eur J Hum Genet.

[58] Dufrasne S, Roy M, Galvez M, Rosenblatt DS (2011). Experience over fifteen years with a protocol for predictive testing for Huntington disease. Mol Genet Metab.

[59] Benatar M, Stanislaw C, Reyes E, Hussain S, Cooley A, Fernandez MC (2016). Presymptomatic ALS genetic counselling and testing: experience and recommendations. Neurology..

[60] Tibben A, Stevens M, de Wert GM, Niermeijer MF, van Duijn CM, van Swieten JC (1997). Preparing for presymptomatic DNA testing for early onset Alzheimer’s disease/cerebral hemorrhage and hereditary Pick disease. J Med Genet.

[61] Trembath MK, Tassicker RJ, Collins VR, Mansie S, Sheffield LJ, Delatycki MB (2006). Fifteen years of experience in predictive testing for Huntington disease at a single testing center in Victoria. Australia Genet Med.

[62] van der Steenstraten IM, Tibben A, Roos RA, van de Kamp JJ, Niermeijer MF (1994). Predictive testing for Huntington Disease: nonparticipants compared with participants in the Dutch program. Am J Hum Genet.

[63] Taylor S (2005). Gender differences in attitudes among those at risk for Huntington’s disease. Genet Test.

[64] Redondo-Sendino A, Guallar-Castillón P, Banegas JR, Rodríguez-Artalejo F (2006). Gender differences in the utilization of health-care services among the older adult population of Spain. BMC Public Health.

[65] Bateman RJ, Benzinger TL, Berry S, Clifford DB, Duggan C, Fagan AM (2017). The DIAN-TU next generation Alzheimer’s prevention trial: adaptive design and disease progression model. Alzheimers Dement.

[66] Roberts JS, LaRusse SA, Katzen H, Whitehouse PJ, Barber M, Post SG (2003). Reasons for seeking genetic susceptibility testing among first-degree relatives of people with Alzheimer disease. Alzheimer Dis Assoc Disord.

[67] Mandich P, Jacopini G, Di Maria E, Sabbadini G, Abbruzzese G, Chimirri F (1998). Predictive testing for Huntington’s disease: ten years’ experience in two Italian centres. Ital J Neurol Sci.

[68] Broadstock M, Michie S, Marteau T (2000). Psychological consequences of predictive genetic testing: a systematic review. Eur J Hum Genet.

[69] Decruyenaere M, Evers-Kiebooms G, Boogaerts A, Cassiman JJ, Cloostermans T, Demyttenaere K (1996). Prediction of psychological functioning one year after the predictive test for Huntington’s disease and impact of the test result on reproductive decision making. J Med Genet.

[70] Meiser B, Dunn S (2000). Psychological impact of genetic testing for Huntington’s disease: an update of the literature. J Neurol Neurosurg Psychiatry.

